# Time resolved structural dynamics of butadiyne-linked porphyrin dimers

**DOI:** 10.1063/1.4940222

**Published:** 2016-01-14

**Authors:** Franco V. A. Camargo, Christopher R. Hall, Harry L. Anderson, Stephen R. Meech, Ismael A. Heisler

**Affiliations:** 1 School of Chemistry, Norwich Research Park, University of East Anglia, Norwich NR4 7TJ, United Kingdom; 2 CAPES Foundation, Ministry of Education of Brazil, Brasilia DF 70040-202, Brazil; 3 Department of Chemistry, University of Oxford, Chemistry Research Laboratory, Oxford OX1 3TA, United Kingdom

## Abstract

In this work, the timescales and mechanisms associated with the structural dynamics of butadiyne-linked porphyrin dimers are investigated through time resolved narrowband pump/broadband probe transient absorption spectroscopy. Our results confirm previous findings that the broadening is partly due to a distribution of structures with different (dihedral) angular conformations. Comparison of measurements with excitations on the red and blue sides of the Q-band unravel the ground and excited state conformational re-equilibration timescales. Further comparison to a planarized dimer, through the addition of a ligand, provides conclusive evidence for the twisting motion performed by the porphyrin dimer in solution.

## I. INTRODUCTION

Conjugated molecular structures composed of porphyrin chromophores have been proposed for use in a wide range of applications such as, for example, novel optical materials for improved solar cells, nanowires for molecular electronics, and in the development of efficient artificial light harvesting materials.^1–8^ Indeed, great progress has been made towards synthesizing conjugated porphyrin multi-chromophore structures tailored specifically for energy materials related applications.^5^ However, extended chromophoric structures inevitably present conformational heterogeneity that can act as an energy or charge sink. This is detrimental to the efficient operation envisaged for photonic wires and biomimetic light harvesting applications.^9,10^

The amount of conformational heterogeneity is affected by the manner in which porphyrin rings are connected (to each other) and by the addition of substituents which can enhance steric hindrance and therefore restrict structural movements.^11^ However, this also restricts the conjugation length and therefore is not the best solution for certain applications.^12^ For an efficient “through bond” charge transport, it is preferable that the porphyrin rings show an extended π-orbital communication (conjugation).^13^ By using different linking moieties, such as vinylene or ethynylene groups, for example, different degrees of conjugation can be achieved. However, linking groups tend to decrease the relative twisting barrier of the porphyrin rings, allowing rotation around the central axis. The rotation modifies the conjugation length, which may enhance or reduce excitation delocalization along the porphyrin oligomer structure.

Detailed information about time resolved structural dynamics in conjugated molecular structures has been obtained mostly through ultrafast spectroscopy methods relying on absorption, fluorescence, and anisotropy observables.^14–16^ Recent experimental developments, such as 2D electronic and IR spectroscopy as well as a phase-modulation fluorescence approach to 2D electronic spectroscopy, started to provide a detailed understanding of the molecular structural dynamics and its effect on the excitonic coupling as a function of different structural conformations.^17–20^

The butadiyne-linked porphyrin structures studied here present inter-porphyrin conjugation as evidenced by a large red shift of the lowest singlet absorption band (Q-band).^21^ However, as expected, the degree of conjugation is significantly affected by the dihedral angle between the porphyrin macrocycles. The maximum conjugation (largest red shift) occurs when the rings are co-planar.^21^ It has been shown previously through a diverse set of steady-state, time resolved spectroscopy, and calculations that there exists a continuous distribution of dihedral angles (in the ground state), indicating that the butadiyne-linker provides a low energy barrier to the rotation of the porphyrin rings.^22^ An unambiguous proof of structural heterogeneity present in butadiyne-linked porphyrin dimers was recently provided by two-dimensional electronic spectroscopy (2D ES) measurements.^23^ The stretched diagonal amplitude distribution at early times observed in the 2D ES measurements corresponds to an inhomogeneously broadened lineshape, which can be assigned to the absorption of a distribution of conformers with different dihedral angles. Further, those measurements provided the rate constants for the twisting reaction in the ground and excited states. However, for the 2D ES measurements, all pulses were degenerate (same spectral composition) therefore restricting measurements to a relatively narrow observation spectral region (typically 2000 cm^−1^). Hence, the published 2D ES studies can be further complemented through the investigation of the molecular response over a broad spectral region, following selective excitation at different absorption bands.

In order to isolate the dynamical evolution of a specific distribution of conformational populations, in this work, we perform narrowband pump/broadband probe transient absorption which is analogous to “hole burning” spectroscopy.^24^ For the butadiyne-linked porphyrin dimers studied here, excitation on the red side of the absorption spectrum, corresponding to excitation of planar conformers, allows the observation of mostly ground state re-equilibration, whereas excitation on the blue side accesses mostly re-equilibration in the excited state. The broadband probe is obtained by white light continuum generation, which allows observation of a wide spectral region covering most of ground state as well as excited state absorption (ESA) of reactant and product states.^25^ The study is complemented by comparisons made between the free dimer and the dimer with an additional ligand connecting the centers of the porphyrin ring, therefore suppressing the twisting motion. Further viscosity and temperature dependent measurements allow the assessment of the solvent friction effect on the porphyrin dimer structural motions.

## II. EXPERIMENTAL METHODS

The transient absorption experiment, which is based on a setup similar to one used by Riedle,^25^ is driven by an amplified titanium:sapphire laser system (Spectra Physics Spitfire ACE) that produces 120 fs pulses at 800 nm with a repetition rate of 1 kHz. The amplified laser pumps two commercial optical parametric amplifiers (Spectra Physics TOPAS Prime). One OPA generates pump-pulses for the sample excitation (70–80 fs pulse duration) at various spectral positions across the visible spectral region. The second OPA is used as a pump for the generation of white light continua in a sapphire window. In this work, the second OPA pump pulses were centred at 1200 nm, producing a probe spectrum spanning the region of 500–900 nm. The pump and probe pulses were focused at the sample position by reflective optics to spot sizes (FWHM) of 170 *μ*m and 30 *μ*m, respectively. For all measurements, the excitation pulse energy was ≤100 nJ, equating to an excitation intensity of 0.4 Wcm^−2^. A waveplate and a polarizer were used to set the polarization of the pump beam at the magic angle, 54.7°, relative to the probe beam. The pump beam was chopped at 500 Hz, with pump-on/pump-off difference spectra calculated for sequential pairs of pulses. A probe reference spectrum was recorded at 1 kHz and was used to correct for fluctuations in the probe spectrum spectral intensity. The presented data are the average of 10 scans, each of which are made up of 500 difference spectra recorded at each delay point. With these acquisition parameters, we can achieve a RMS noise level better than 50 μOD. The probe spectrum was dispersed using a home built prism spectrometer with a spectral resolution of 3 nm. The time resolution of the experiment was determined to be 150 fs through measurements performed in pure solvent (width of “coherence spike” around delay time zero).

**FIG. 1.**
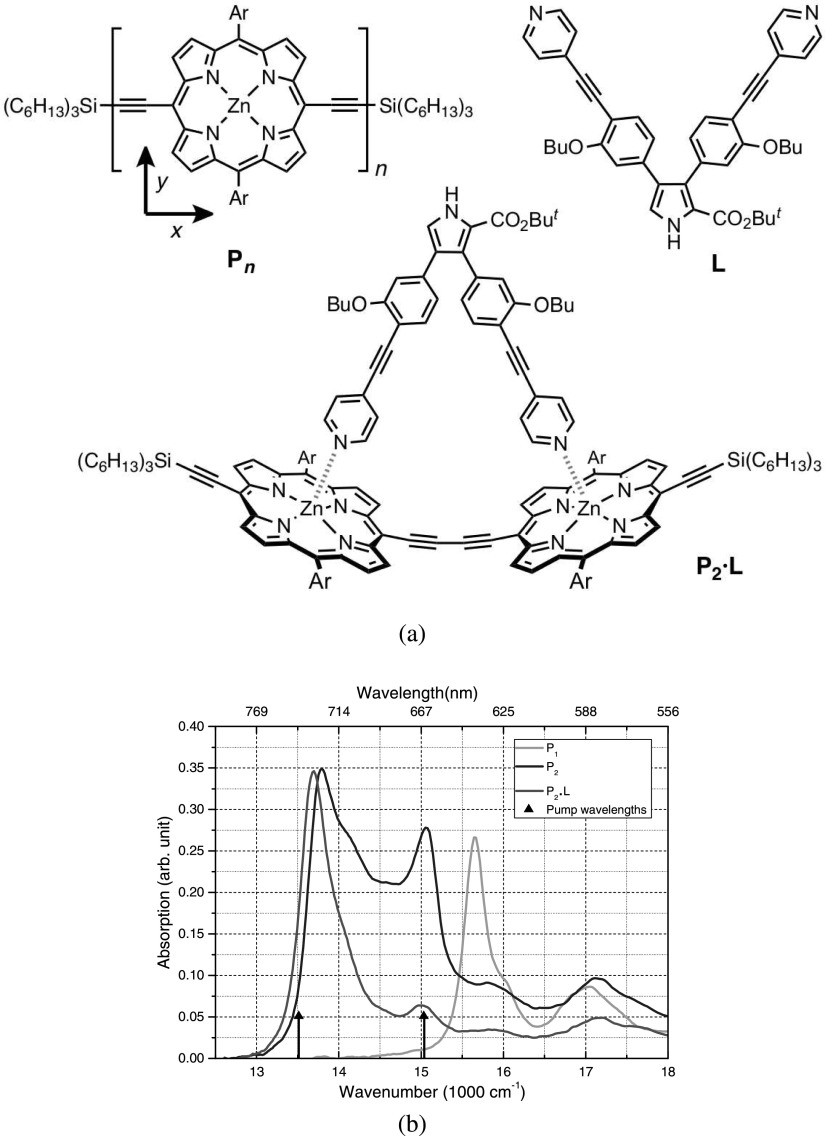
(a) Porphyrin monomer, dimer and ligand structures where Ar = 3,5-bis(octyloxy)phenyl. (b) Q-band region linear absorption spectrum of the porphyrin monomer (green line), dimer (blue line), and dimer with ligand (red line) in pentane containing 1% pyridine at 298 K.

The porphyrin structures, shown in Figure 1(a), were synthesized as reported previously.^26^ All porphyrin structures were dissolved in alkane solvents (pentane, heptane, octane, and decane) as well as in toluene, with typical concentrations of 50 *μ*M for a 1 mm pathlength static cell producing an OD around 0.3 in the Q-band region. In order to avoid aggregation, 1 vol.% pyridine was added to the solution.

## III. RESULTS AND DISCUSSION

### A. Steady state absorption and fluorescence spectra

In order to better understand the dimer linear absorption spectrum, we start by describing the assignment of the main electronic transitions present in the parent monomer, P_1_, whose molecular structure is shown in Figure 1(a) (P_n_—with n = 1). The visible to near UV absorption spectrum of P_1_ is shown in Figure S1(a) (supplementary material).^27^ Similarly to other substituted porphyrin structures, the lowest energy spectral amplitude is related to transitions from the ground state into the so called Q-band.^3^ Due to the asymmetric substituents present in P_1_, the degeneracy of the transition dipole moments along the x and y axes is lifted (axes are defined in Figure 1(a)). The lowest energy transition corresponds to the transition dipole moment along the axis containing the acetylene groups and is defined as the x-axis. Therefore, the Q_x_ transition is assigned to the peak at 15 650 cm^−1^, whereas the Q_y_ transition is assigned to the peak at 16 950 cm^−1^. Differently from the more widely studied zinc tetraphenylporphyrin (ZnTPP), for P_1_, the Q_x_ (0,0)) transition is stronger than its vibronic counterpart, Q_x_ (0,1).^28^ The shoulder at 16 030 cm^−1^ is assigned to a 380 cm^−1^ vibrational mode strongly coupled to the Q_x_ transition.^29^ Another vibrational mode (1340 cm^−1^) also couples to the Q_x_ transition producing a shoulder at 16 990 cm^−1^ and, therefore, overlapping with the Q_y_ transition. The monomer P_1_ fluorescence spectrum is shown in Figure S1(b) together with its linear absorption spectrum.^27^ As in other porphyrin molecules, P_1_ fluorescence has only a small Stokes shift (≈50 cm^−1^) and mirrors quite well its linear absorption spectrum.

The second excitation appears at 22 000 cm^−1^ and is much stronger than the Q_x_ transition. Similarly to other porphyrin structures, this band is usually called the Soret or B-band.^3^ Furthermore, another broad high energy band is located above 27 000 cm^−1^ and known as N-band (Figure S1(a)).^27^ Those bands are also present in the dimer P_2_ and the dimer planarized with the ligand, P_2_·L, whose structures are shown in Figure 1(a). The dimerization produces a splitting of the Soret band and induces a broadening and red shift of the linear absorption spectrum in the Q-band region. The observed large red shift of the lowest singlet absorption band (corresponding to the Q_x_ transitions) occurs due to the butadiyne linker inter-porphyrin conjugation, which leads to a stabilization of the excited state in the dimer.^22^ However, the amount of conjugation or electronic coupling induced by the butadiyne linker is significantly affected by the dihedral angle between the porphyrin macrocycles. Therefore, maximum coupling (largest red shift) occurs when the rings are co-planar. Due to a low energetic barrier, a distribution of conformations of different dihedral angles is expected in the ground state with a consequent broadening of the linear absorption spectrum. It was previously suggested that this spectral region comprises absorption of the planar (0°) and fully twisted (90°) conformers of the dimer at 13 513 cm^−1^ (740 nm) and 14 948 cm^−1^ (669 nm), respectively.^22^ Further contributions to the linear absorption spectrum are due to vibronic transitions (also present in P_1_) that appear as shoulders at 13 884 cm^−1^ (380 cm^−1^ vibrational mode), at 14 333 cm^−1^ (825 cm^−1^ vibrational mode), and at 14 863 cm^−1^ (1340 cm^−1^ vibrational mode). An interesting feature is the enhancement of the 825 cm^−1^ vibrational mode in the dimer. This could be due to the fact that the butadiyne-linker promotes a stronger coupling of this vibrational mode to the Q_x_ electronic transition.

A first assessment of the dimer structural heterogeneity can be obtained by using a ligand that connects to the dimer in such a way as to impose dihedral planarity. This can be achieved by the addition of a bidentate dipyridyl pyrrole ligand L (Figure 1(a)). As shown before, the ligand L forms a strong 1:1 complex with P_2_ (this structure is labeled as P_2_·L) and assumed to induce a dimer dihedral angle close to zero.^22^ The main alterations observed in the linear absorption spectrum by the addition of L to P_2_ can be summarized as follows: in the Soret band, an amplitude decrease of the peak around 21 882 cm^−1^ (457 nm) and a concomitant amplitude increase of the peak at 20 366 cm^−1^ (491 nm) are observed whereas in the Q-band the broad spectral region around 15 037 cm^−1^ (665 nm) decreases in amplitude with a concomitant amplitude increase of the band around 13 513 cm^−1^ (740 nm). Therefore, the lowest energy peak in the Soret band at 20 366 cm^−1^ and in the Q-band at 13 513 cm^−1^ can be associated to planar conformations, whereas the higher energy peak in the Soret band at 21 882 cm^−1^ and in the Q-band at 15 037 cm^−1^ correspond to higher dihedral angular conformations. This has been confirmed by steady state excitation spectra reported earlier.^22^ Steady state fluorescence also provides some preliminary evidence for planarization of P_2_ in the excited state. Figures S1(c) and S1(d), respectively, show the steady state fluorescence spectra for P_2_ and P_2_·L, for an excitation at 20 284 cm^−1^ (493 nm), together with their respective linear absorption spectra. The fluorescence spectrum of P_2_ has great resemblance to P_2_·L fluorescence spectrum meaning that only fluorescence from the lowest energy stabilized structure is detected, which was shown earlier to correspond planar porphyrin dimers.

### B. Time resolved transient absorption spectra

**FIG. 2.**
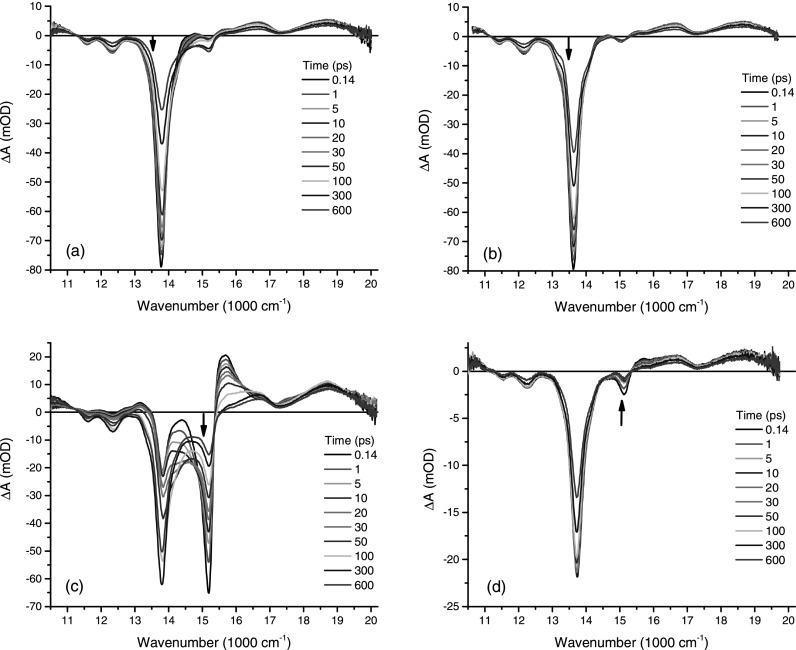
Time resolved transient absorption spectra for times ranging from 0.14 to 600 ps as shown on each graph. The results are shown for the porphyrin dimer without ligand (a) with pump excitation at 13 513 cm^−1^ and (c) for pump excitation at 15 037 cm^−1^. The porphyrin dimer with ligand is shown in (b) for pump excitation at 13 513 cm^−1^ and (d) for pump excitation at 15 037 cm^−1^. The pump excitation wavenumber is marked by a black arrow in each graph.

Detailed information about the spectral composition and dynamical evolution underlying the linear spectra of P_2_ and P_2_·L was obtained by narrow band pump–broadband probe transient absorption measurements. Figure 2(a) presents a series of time resolved transient absorption spectra (0.14 ps to 600 ps) for P_2_ dissolved in pentane (containing 1% pyridine at 298 K) for an excitation at 13 513 cm^−1^ (740 nm—black arrow). This excitation, on the red side of the maximum of the Q-band absorption, provides little or no excess vibrational energy and is ideally suited to probe structural and solvation dynamics. The resulting probe absorption change comprises a strong ground state bleach (GSB) of the Q_x_(0,0) band (plus vibronic shoulder, Q_x_(0,1)) as well as stimulated emission (SE) in the region from 12 864 cm^−1^ to 15 570 cm^−1^. For wavenumbers below 12 864 cm^−1^, the negative signal is assigned to SE from the Q_x_(0,1) vibronic transitions corresponding to a 1340 cm^−1^ vibrational mode (detected at 12 333 cm^−1^) and another 2218 cm^−1^ vibrational mode (detected at 11 550 cm^−1^). The 2218 cm^−1^ vibrational mode matches the carbon-carbon triple (C≡C) bond stretching frequency.^30^ Consequently, the band appearing at 15857 cm^−1^ in the linear absorption spectrum of P_2_ and P_2_·L can be assigned to a vibronic shoulder due to the coupling of the C≡C stretching mode to the Q_x_ transition, which is to be expected given that the transition dipole moment is along the butadiyne linker where two C≡C moieties appear. Further, in Figure 2(a), a broad ESA appears for wavenumbers above 15 350 cm^−1^ and below 11 200 cm^−1^. This broad ESA is a well known feature present in porphyrin transient absorption spectra and has been assigned to singlet (S_1_ => S_n_) as well as triplet (T_1_ => T_n_) transitions.^15,31^

The ground state structural re-equilibration is captured by the GSB amplitude increase in the wavenumber region around 15 000 cm^−1^ (Figure 2(a)). This spectral feature can be interpreted as an indication that the pump beam, with a bandwidth of 190 cm^−1^, carves a hole in the ground state population conformational distribution creating, at the same time, an excited state population with a narrow dihedral angle distribution. Given that only the lowest energy (quasi-planar) conformers are excited and the fact that the barrier to twist in the excited state is significantly above *k*T at 298 K, the ground state twisting re-equilibration within the thermal distribution is observed. The net result of this re-equilibration is the appearance of porphyrin dimers with non-zero dihedral angles, leading to a bleach increase in the spectral region associated with twisted conformers. This interpretation was confirmed by carrying out the same measurements for the P_2_·L structure, as shown in Figure 2(b). The main difference between the P_2_ and P_2_·L time resolved transient absorption spectra is the absence of GSB amplitude decrease at 13 810 cm^−1^, with a concomitant amplitude increase at 15 000 cm^−1^, observed in P_2_.

**FIG. 3.**
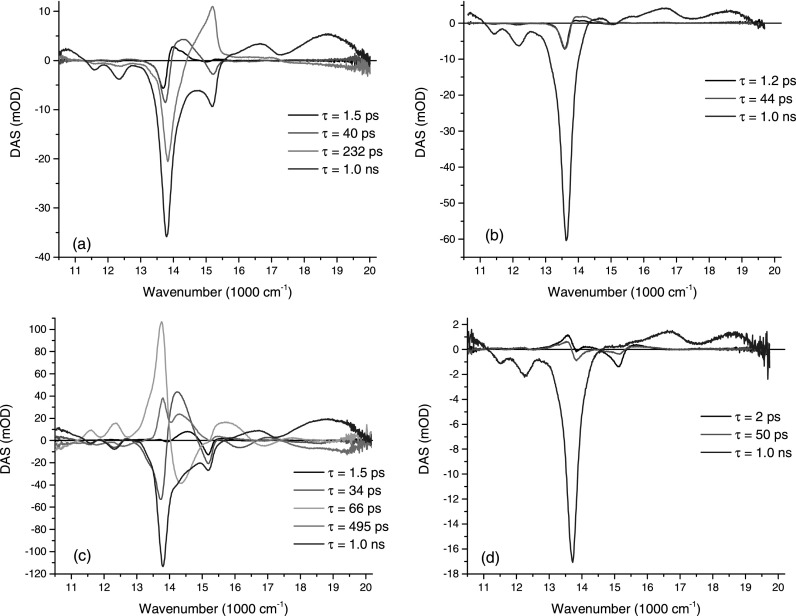
Decay associated spectra (DAS) with their corresponding exponential time constants. The results are shown for the porphyrin dimer without ligand (a) with pump excitation at 13 513 cm^−1^ and (c) for pump excitation at 15 037 cm^−1^. The porphyrin dimer with ligand is shown in (b) for pump excitation at 13 513 cm^−1^ and (d) for pump excitation at 15 037 cm^−1^.

The lifetimes associated with rising and decaying contributions to the time resolved spectra were obtained by applying a global analysis fitting, where the time resolved spectra (as a function of population time) are fit to a single model consisting of a multi-exponential relaxation.^32^ For the excitation on the red side of the absorption spectrum (13513 cm^−1^), four exponential contributions were necessary to fit the data properly. When fitting the data in this manner, a parallel decay mechanism is assumed. The pre-exponential factors (exponential amplitudes) retrieved from the fit are called decay associated spectra (DAS). It is also possible to assume a sequential model (where one spectrum turns into another sequentially) which can be calculated from the originally recovered DAS. The evolutionary associated spectra (EAS) thus obtained are shown (for completeness) in Figure S2. Here, we will focus on the retrieved DAS for P_2_ and P_2_·L, which are shown in Figures 3(a) and 3(b), respectively. A negative (positive) DAS in a region were the measured signal has negative (positive) amplitude translates into a decaying (rising) exponential contribution. For positive measured signals, the assignment is exactly the opposite, i.e., a negative (positive) DAS translates into rising (decaying) exponential contributions. The shortest DAS component, with a lifetime of τ = 1.5 ps, can be assigned to relatively fast local population re-equilibration processes such as intramolecular vibrational energy redistribution (IVR), vibrational cooling (VC), or solvation dynamics, although the latter is unlikely given that the solvent was the nonpolar and low viscosity pentane. This component has a similar shape in both P_2_ and P_2_·L, corresponding to a negative DAS in the spectral position where the pump beam is located and positive DAS amplitude towards higher frequencies, corresponding to a slight spectral broadening and blue shifting. A similar time constant was revealed previously by 2D ES measurements, corresponding to an overall broadening of the 2D spectra over a picosecond timescale.^23^ It was argued that it could be connected to an overdamped oscillation of a torsional motion of the dimer. On the other extreme, the longest DAS contribution, with a time constant of τ = 1 ns (which compares well with the 1.2 ns porphyrin dimer fluorescence lifetime reported in literature), can be associated with the population relaxation of the first singlet excited band and corresponding ground state bleach recovery of the structurally relaxed porphyrin dimer.^22^ This DAS component shows a decaying GSB + SE (negative DAS over a negative signal region) with a concomitant ESA decay (positive DAS over a positive signal region). The next shortest DAS contribution, with a time constant τ = 40 ps, appears in both P_2_ and P_2_·L and has a similar shape to the short τ = 1.5 ps, although its amplitude is enhanced for P_2_. The 40 ps relaxation is too slow to be ascribed to relaxation mechanisms such as IVR, VC, or solvation and therefore we delay the discussion of this component for later in this paper when more results will be presented and discussed. The final DAS contribution, with a lifetime of τ = 232 ps, most clearly captures the amplitude decrease on the red side (with a concomitant amplitude increase on the blue side) of the GSB + SE, in the region 13 100 < $\bar{\upsilon }\;$ < 15 570 cm^−1^. Given the timescale of this DAS component and the fact that it is absent in the retrieved DAS for P_2_·L (Figure 3(b)), it can be safely assigned to a major structural relaxation mechanism and which, for this dimer, can be assigned to the twisting motion of the porphyrin rings around the central butadiyne linking axis. This motion is suppressed by the addition of the ligand to the porphyrin dimer, as shown by the measurements obtained for P_2_·L. Further, the τ = 232 ps DAS component has negligible amplitude for spectral regions outside 13 100 < $\bar{\upsilon }\;$ < 15 570 cm^−1^, corresponding to SE and ESA contributions. This is a clear indication that the structural re-equilibration accessed by this DAS component indeed happens in the ground state. To confirm that the minor amplitude found for this component, in the ESA and SE regions outside 13 100 < $\bar{\upsilon }\;$ < 15 570 cm^−1^, was not crucial, individual time traces were fit with and without the presence of this extra time component. The results reproduced the same time constants for all the other exponential terms regardless of whether the extra component was included, in which case the fit was only marginally better.

**FIG. 4.**
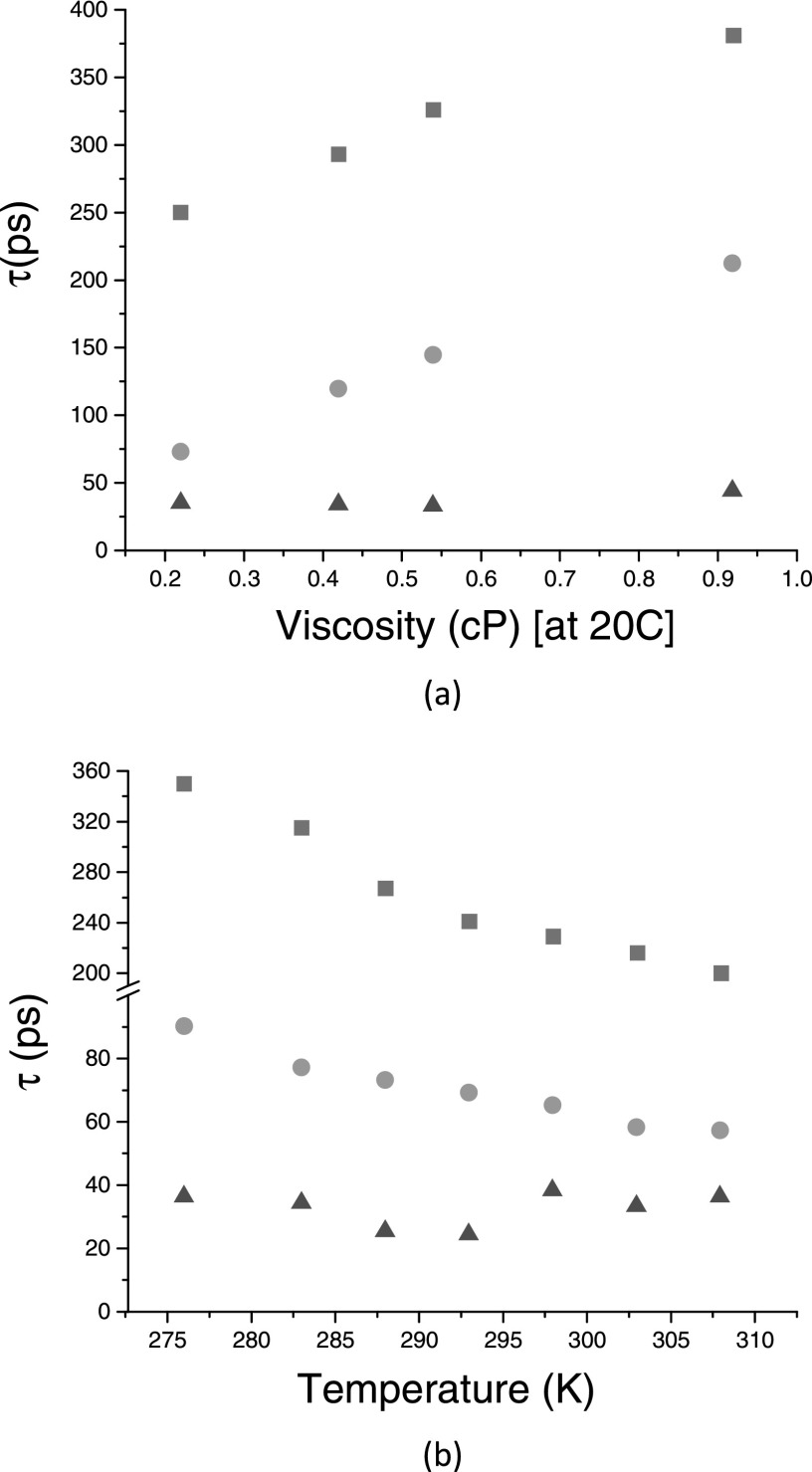
Ground state (dark yellow squares) and excited state (green circles) twisting motion time constants as a function of (a) viscosity and (b) temperature. The red triangles in (a) and (b) correspond to the time constants associated to the second DAS (red curve in Figure 3(c)) retrieved for P_2_ for an excitation at 15 037 cm^−1^, as a function of temperature.

The time constant, τ = 232 ps, agrees well with the similar time constant obtained previously with 2D ES measurements over a narrower spectral region and which was assigned to the ground state porphyrin dimer twisting re-equilibration.^23^ The assignment of this relaxation mechanism to the evolution in dihedral angle requires the movement of a bulky molecular structure and, therefore, it is anticipated that friction from a viscous solvent should affect the rate of this motion. This was tested by performing measurements in a series of alkane solvents as well as in toluene and the retrieved time constants are shown in Figure 4(a) (dark yellow squares). As expected, a viscosity increase translates into a slower twisting motion. The same effect (twisting motion slowdown) is seen by lowering the temperature as shown in Figure 4(b) (dark yellow squares).

Although the rationale for understanding the retrieved DAS was laid out above, a better visualization of the dynamics is provided by time resolved curves for specific spectral positions. Figure S3 compares curves for P_2_ and P_2_·L for four different spectral positions marked A to D which capture the main spectral dynamical evolution.^27^ The P_2_ and P_2_·L comparison curves obtained for positions A and D, associated with the red side SE and blue side ESA, respectively, very clearly relax with similar time constants. We also fitted those curves independently to confirm the time constants obtained by the global analysis fitting procedure. The structural re-equilibration is most clearly captured by spectral positions B and C. Regarding spectral position C, the time resolved GSB + SE amplitude for P_2_·L shows a monotonic decay whereas for P_2_ the negative signal increases (becomes more negative) as reflected by the positive DAS for this spectral region shown in Figure 3(a). Regarding spectral position B, P_2_ shows a clear extra GSB + SE amplitude decrease when compared with P_2_·L and as reflected in the negative DAS for this spectral region shown in Figure 3(a).

**FIG. 5.**
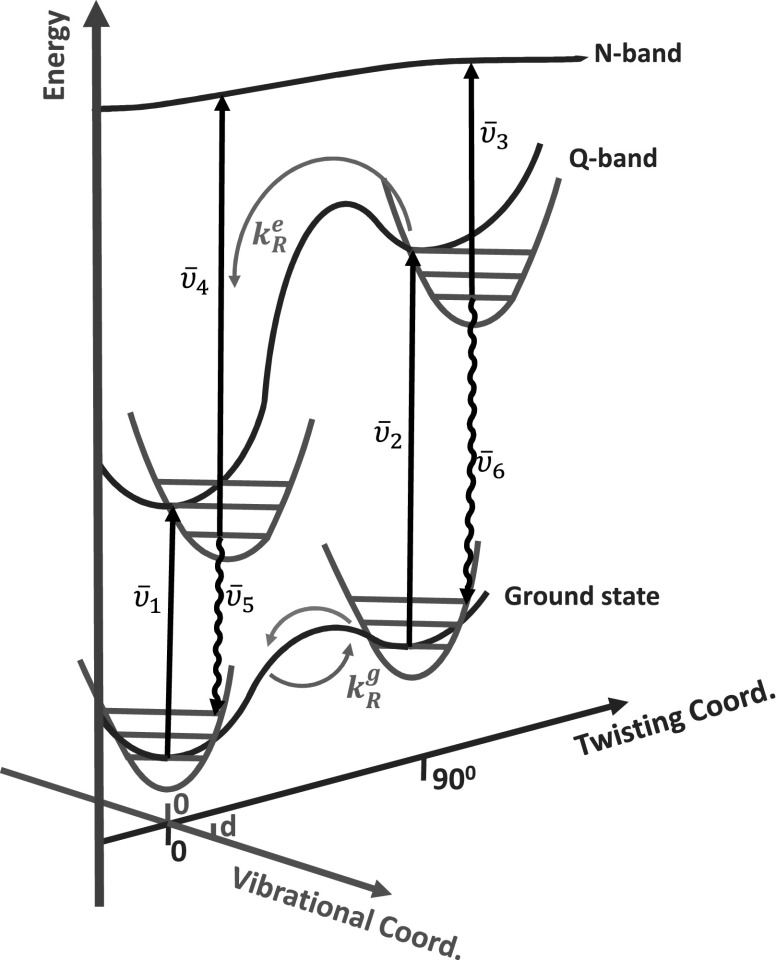
Schematic representation of the porphyrin dimer potential energy surface (PES) as a function of the twisting and vibrational coordinates. The constants $k_{R}^{e}\;$ and $k_{R}^{g}$ represent the excited state and ground state twisting reaction rates, respectively. In the excited state, due to a high energy barrier, only the planarization reaction is observed, whereas in the ground state forward and reverse reactions are detected.

The results obtained so far can be understood in terms of a potential energy surface (PES) outlined in Figure 5. Excitation on the red edge, corresponding to ${\bar{\upsilon }}_{1}$ = 13513 cm^−1^, does not provide enough energy for the molecule to overcome the barrier in the excited state which was estimated previously by Winters *et al*. through quantum mechanical calculations to be about 6 *k*T at 298 K.^22,33^ The same calculations indicated that the ground state PES exhibits a low barrier for rotation (∼*k*T) and should thus have a broad distribution of dihedral angles at room temperature. Indeed, the broad ESA, corresponding to ${\bar{\upsilon }}_{4}$ > 15570 cm^−1^, does not show any temporal evolution apart from an overall population decay on a nanosecond timescale (Figures 2(a) and 3(a)). The only spectral evolution observed for this low energy excitation is a bleach rebalancing on a timescale of τ = 232 ps, in agreement with our previous 2D ES measurements.

In order to access the excited state structural dynamics, the pump wavelength was tuned to the blue of Q-band absorption peak to excite specifically the band at 15 037 cm ^− 1^ (665 nm). As discussed above, this spectral region was assigned to non-planar dimer conformations. The time resolved absorption spectra for this excitation wavelength are shown in Figure 2(c). The time evolution is significantly different when compared with the spectra obtained by exciting the low energy edge of the Q-band. The main new features can be summarized as follows. An extra ESA band appears at 15 624 cm^−1^. This band appears within the time resolution of our experiment (150 fs) and decays on a 70 ps timescale decay. The spectral region related to the SE from the vibronic peaks, between 11 200 cm^−1^ and 13 550 cm^−1^, starts off from zero and increases in amplitude (signal becomes more negative) on the same 70 ps timescale as observed for the extra ESA decay. The GSB band at 15 100 cm^−1^ decays monotonically, whereas the GSB + SE band at 13 750 cm^−1^ rises on a 70 ps timescale and finally decays on a longer timescale. The ESA and SE spectral features unambiguously point to an excited structural re-equilibration. The dynamics revealed by the spectral evolution shown in Figure 2(c) is that of a planarization of dihedrally distorted conformers, mainly on a timescale of 70 ps. Again, this can be further confirmed by performing the same measurements on P_2_·L, as shown in Figure 2(d). The difference in spectral evolution is striking. Apart from an extra bleach contribution around the excitation wavenumber (at 15 037 cm^−1^), the P_2_·L time resolved spectra are very similar to the spectra obtained by excitation at low energies. This points to the fact that also in the excited state structural heterogeneity is strongly suppressed by the addition of the ligand.

The lifetimes associated with the time resolved spectra for excitation at 15 037 cm^−1^ were also obtained through a global analysis fitting procedure, although five exponential terms were necessary to properly fit the data. The retrieved DAS curves are shown in Figure 3(c). The two fastest DAS curves (τ = 1.5 ps and τ = 33 ps) have similar shapes and time constants to the equivalent DAS components retrieved for low energy excitation and can, therefore, be assigned to similar relaxation mechanisms. This is also true for the slowest contribution with a time constant of τ = 1 ns. The DAS contribution with a time constant τ = 495 ps has a different shape and no immediate equivalent to any DAS contribution retrieved for low energy excitation. Although it is expected that for excitation at 15 037 cm^−1^, the twisting dynamics in the ground state should also occur with the same timescale (related to the inverse of the twisting reaction) and leave a DAS signature. However, for this excitation, many overlapping features, including the much more intense planarization in the excited state, hinder a clear identification of this ground state contribution. The τ = 495 ps DAS shows essentially no changes in the region around 14 948 cm^−1^ (related to the twisted conformers), accompanied by a clear rise at smaller wavenumbers all the way through to 13 500 cm^−1^ (small dihedral angles related to planar conformers), which is consistent with the planarization of molecules initially excited in the twisted conformation. The presence of amplitude in the wavenumber region above 17 500 cm^−1^ (where ESA dominates) can be considered as a fitting artifact, as in this region the two dominating DAS components are the 495 ps and the 1 ns, and there might be some interplay between them.

The final τ = 66 ps DAS, which also has no equivalent in the previously retrieved DAS for low energy excitation, can be unambiguously assigned to the excited state porphyrin dimer planarization. This curve assigns a positive amplitude for the two vibronic peaks as well as for the extra ESA signal at around 15 624 cm^−1^, meaning that the negative SE signal rises, whereas the positive ESA signal decays with this timescale. Furthermore, this DAS contribution is absent in the DAS retrieved for the P_2_·L measurements, as shown in Figure 3(d). Again, a better visualization of the dynamics is provided by time resolved curves for specific spectral positions. Figure S4 shows the comparisons between P_2_ and P_2_·L for time resolved measurements for five different spectral positions marked A to E, which capture the main spectral dynamical evolution. Compared with P_2_·L, the P_2_ curves for positions A and B show a clear rising signal (up to 200 ps) followed a slower decay. The extra ESA decay, captured by graph D (and absent for P_2_·L), is matched by a rising component in the ESA spectral region associated to the planar conformations, corresponding to graph E, and only present in P_2_. Similarly, to the ground state twisting motion, the excited state planarization slows down with increasing solvent viscosity and decreasing temperature, as shown in Figures 4(a) and 4(b) (green circles), respectively. This is evidence that the molecular structural motion in the excited state has a similar origin (twisting around porphyrin dihedral angle) because it is equally sensitive to solvent friction as the motion in the ground state. However, in the excited state, the steep potential energy offers a significant driving force accelerating the twisting motion considerably when compared with the ground state. The timescale for this structural motion is in good agreement with previously retrieved times obtained with 2D ES spectroscopy.^23^ From the 2D ES measurements, it was clearly shown that the reverse motion in the excited state was almost completely suppressed due to a high barrier. Also, in this work, the transient absorption data do not provide any evidence for the uphill reverse twisting motion. Unfortunately, time resolved measurements, such as transient absorption or 2D ES, are not able to provide information about the exact dihedral angular displacement that the porphyrin dimer undergoes during its relaxation. Those measurements tend to quantify the amount of a given species present in solution, for a given excitation/detection spectral region. Therefore, information about the width of the dihedral distribution can be gathered by selectively exciting P_2_ towards higher energies. Such measurements are shown in Figure S5, where we progressively increase the pump energy from 14 084 cm^−1^ up into the region of the Q_y_ band at 17 000 cm^−1^.^27^ As discussed above, the clearest marker of the presence of dihedrally twisted conformers in the excited state is provided by the ESA band at 15 660 cm^−1^. A small ESA contribution can already be identified for the lowest excitation energy (14 084 cm^−1^), whereas this contribution increases significantly when exciting at 15 037 cm^−1^. Even for excitation energies as high as 17094 cm^−1^, a significant population of twisted conformers can be excited. When moving into the Soret band, the excitation conditions reproduce the spectra observed for excitation in the Q_x_-band (data not shown) pointing to the fact that indeed the Soret band allows selective excitation of planar and twisted conformers.

Again, the findings so far can be understood in the context of the PES shown in Figure 5. The blue side excitation, corresponding to ${\bar{\upsilon }}_{2}$ = 15037 cm^−1^, excites preferentially non-planar conformations. Due to the flatness of the high energy N-band, the non-planar porphyrin dimer ESA (${\bar{\upsilon }}_{3}$ in Figure 5) appears in a region around 15 715 cm^−1^. Therefore, a strong driving force, due to a steep potential, drives the porphyrin dimer towards planarization. The immediate effect of this planarization is decay of the ESA at 15715 cm^−1^ with a concomitant rise of ESA in the planar region as observed in the time resolved spectra and retrieved DAS (Figures 2(c) and 3(c)). In our PES scheme, the ESA of the less distorted conformers correspond to ${\bar{\upsilon }}_{4}$ = 18660 cm^−1^. The addition of the ligand to the porphyrin dimer most probably has the effect to increase the energy barrier between planar and non-planar conformations, already in the ground state.

Finally, we would like to discuss the yet unassigned second shortest DAS component present in all samples studied and for all excitation wavenumbers. Time constant for this relaxation component is typically ≈40 ps. As shown in Figure 4(b) (red triangles), this contribution is insensitive to temperature changes, distinguishing it from the twisting related time constants which are clearly affected by temperature changes. Further, this contribution is also insensitive to viscosity changes, as shown in Figure 4(a) (red triangles). This points to the fact that if this relaxation were related to some other structural relaxation mechanism present in the porphyrin dimer, the coordinate along which the relaxation occurs should be volume conserving and solvent friction independent. As discussed above, it is highly unlikely that this contribution should be related to IVR which, for molecules of similar sizes to those studied here, occurs typically on a timescale of a few hundred femtoseconds.^34^ The 40 ps timescale we observed is therefore much too long. On the other hand, it was shown that VC can occur on timescales of tens of picoseconds. However, the relaxation timescale should be solvent dependent as the spectral density of states offered by some solvents can enhance or suppresses VC relaxation. We do not see any solvent dependence for this relaxation timescale, although we might have worked with a too narrow range of solvents. Another possible structural relaxation mechanism present in bridged dimeric structures is given by the bridge elongation/contraction relaxation motion. Relaxation along this coordinate does not entail significant solvent dislocation; therefore, it is insensitive to solvent viscosity. However, without further detailed molecular scale calculations which could help to more clearly identify the electronic transitions contributing to the broad absorption spectra of the butadiyne-linked porphyrin dimers studied here, the final assignment of this contribution to time resolved spectra remains open.

## IV. CONCLUSION

In this work, we presented and discussed the results obtained by narrowband pump/broadband probe transient absorption spectroscopy applied to two sets of butadiyne-linked porphyrin dimers. The use of a narrowband excitation pulses enabled selective observation of structural re-equilibration on the ground and excited state surfaces. Further, comparison to measurements performed on the same dimer but with an added ligand, which suppresses dihedral twisting motion, enabled identification of the structural motion and assignment to the porphyrin dimer twisting motion around the central butadiyne-linking axis. Through global analysis, the retrieved timescales of the structural re-equilibration in the ground and excited states were 232 ps and 66 ps, respectively, at room temperature and in pentane. These time constants are in good agreement with similar timescales obtained previously with 2D ES measurements. A further relaxation mechanism, with a time constant around 40 ps, was identified. Differently from the time constants related to the twisting motion in the ground and excited states, this component is viscosity and temperature independent. Even though it is insensitive to solvent friction, the amplitude of this relaxation contribution is enhanced by the twisting motion, a conclusion obtained by comparing the results for P_2_ and P_2_·L. One line of argument is that this contribution might correspond to the bridge elongation/contraction relaxation motion. However, without further detailed calculations and follow up measurements (such as very low temperature), this assignment remains unclear.
